# Regulation of pre-fusion events: recruitment of M-cadherin to microrafts organized at fusion-competent sites of myogenic cells

**DOI:** 10.1186/1471-2121-14-37

**Published:** 2013-08-27

**Authors:** Atsushi Mukai, Naohiro Hashimoto

**Affiliations:** 1 Department of Regenerative Medicine, National Institute for Longevity Sciences, National Center for Geriatrics and Gerontology, 35 Gengo, Morioka, Oobu, Aichi 474-8522, Japan

**Keywords:** Myogenesis, Cell fusion, M-cadherin, p120 catenin, Rac1, Tyrosine phosphorylation

## Abstract

**Background:**

Previous research indicates that the membrane ruffles and leading edge of lamellipodia of myogenic cells contain presumptive fusion sites. A micrometer-sized lipid raft (microraft) is organized at the presumptive fusion site of mouse myogenic cells in a cell-contact independent way and serves as a platform tethering adhesion proteins that are relevant to cell fusion. However, the mechanisms underlying recruitment of adhesion proteins to lipid rafts and microraft organization remain unknown.

**Results:**

Here we show that small G-protein Rac1 was required for microraft organization and subsequent cell fusion. However, Rac1 activity was unnecessary for recruitment of M-cadherin to lipid rafts. We found that p120 catenin (p120) binds to M-cadherin exclusively in lipid rafts of differentiating myogenic cells. The Src kinase inhibitor SU6656 prevented p120 binding to M-cadherin and their recruitment to lipid rafts, then suppressed microraft organization, membrane ruffling, and myogenic cell fusion. Suppression of membrane ruffling in SU6656-treated cells was partially restored by pretreatment with the protein tyrosine phosphatase inhibitor vanadate. The present analyses using an antibody to tyrosine phosphorylated p120 suggest that Src family kinases play a role in binding of p120 to M-cadherin and the recruitment of M-cadherin to lipid rafts through phosphorylation of putative substrates other than p120.

**Conclusions:**

The present study showed that the procedure establishing fusion-competent sites consists of two sequential events: recruitment of adhesion complexes to lipid rafts and organization of microrafts. The recruitment of M-cadherin to lipid rafts depended on interaction with p120 catenin, whereas the organization of microrafts was controlled by a small G protein, Rac1.

## Background

A skeletal muscle fiber is an extra-large, multinucleated, non-mitotic cells that are responsible for the generation of force by skeletal muscle under the control of motor neurones. This unique terminally differentiated cells are derived from multinucleated myotubes, which are formed by the fusion of mononucleated myogenic progenitor cells (myoblasts). Myoblasts are descendants of muscle stem cells called muscle satellite cells and show unique capacities, including multipotentiality [1] and the ability to fuse with each other in a cell-autonomous way. Myoblast fusion is cell-specific, because myoblasts do not fuse with non-myogenic cells, and essential for skeletal muscle development and repair.

Myoblast fusion consists of a series of steps: cell-cell contact, recognition, adhesion, and plasma membrane breakdown/union [2-4]. Plasma membrane breakdown/union is initially induced in a discrete area of the plasma membrane [5,6]. Thus, specialization of presumptive fusion sites in the plasma membrane is prerequisite for myogenic cell fusion. Extracellular matrix receptor integrins and adhesion molecules such as cadherins, NCAM, CD9, CD81, and ADAMs might contribute to regulation of the recognition/adhesion steps of myoblast fusion [7-9]. However, how they accumulate at the discrete, presumptively fusion-competent sites of the plasma membrane remains to be determined.

Our previous study showed that the leading edge of lamellipodia and membrane ruffles of differentiating myogenic cells contain fusion-competent sites in the plasma membrane [5]. Adhesion proteins accumulate at the presumptive fusion sites of differentiating myogenic cells in a lipid raft-dependent fashion prior to cell contact [10], while membrane fusion takes place within cholesterol-free sites of the plasma membrane [11]. Membrane cholesterol is enriched in lipid rafts. However, these results are not discrepant because dynamic clustering and dispersion of lipid rafts plays a pivotal role in the redistribution of adhesion complexes and membrane cholesterol at the presumptively fusion-competent sites of the plasma membrane in myogenic cells [10]. The adhesion complexes accumulate in a micrometer-scaled lipid raft (microraft) in a cell contact-independent fashion under the differentiation-inducing condition, whereas they are distributed in both raft and non-raft fractions of plasma membranes in growing myogenic cells. Therefore, both the recruitment of adhesion complexes to lipid rafts and the organization of microrafts might be critical to plasma membrane breakdown/union of myogenic cells.

M-cadherin is a myogenic cell-specific classic cadherin that plays a pivotal role in myogenic cell fusion [8,12-16]. Adhesion-complex proteins including M-cadherin, β-catenin, and p120 catenin accumulate in microrafts at presumptive fusion sites even if myogenic cells do not contact a fusion partner [10]. The present study showed that recruitment of M-cadherin/p120 complex to lipid rafts and organization of microrafts are two distinct pre-fusion events that are essential for the specialization of fusion-competent sites.

## Methods

### Cell culture

The mouse myogenic cell clone Ric10 was established from muscle satellite cells of the normal gastrocnemius muscle of an adult female ICR mouse [1,5]. Ric10 cells were plated on dishes coated with type I collagen (Sumilon, Tokyo, Japan) and cultured at 37°C under 10% CO_2_ in pmGM consisting of Dulbecco’s modified Eagle’s medium supplemented with 20% fetal bovine serum (FBS), 2% Ultroser G (Biosepra, Cedex-Saint-Christophe, France), and glucose (4.5 mg/ml) [1,17-19]. For induction of myogenic differentiation, the cells were plated and cultured for 24 h in pmGM, and then the medium was changed to pmDM consisting of the chemically defined medium TIS [20,21] supplemented with 2% FBS. A Ric10-derived clone constitutively expressing GFP-GPI, GGS25 [10], was cultured under the same conditions as Ric10. The Src kinase inhibitor SU6656 and Rac1 inhibitor NSC23766 (Sigma, St. Louis, MO) were dissolved in dimethylsulfoxide and diluted with culture medium immediately before use.

### Transfection

Ric10 cells (2×10^4^ cells in a 35-mm dish) were transfected with 0.9 μg of pcDNA-GFP-Rac1wt, GFP-Rac1DA, and GFP-Rac1DN (kindly provided by K. Kaibuchi, Nagoya University) in the presence of 4.5 μl of FuGENE6 transfection reagent (Roche Diagnostic, Mannheim, Germany) as previously described [20-22].

### Immunofluorescence analyses

Cells were grown on collagen-coated culture dishes, then fixed, permeabilized, and processed for immunostaining as described [1,5]. Primary antibodies included mouse monoclonal antibodies to sarcomeric myosin heavy chain (MyHC) (MF20; undiluted culture supernatant) [23], M-cadherin (1:250 dilution; BD Biosciences, San Jose, CA), p120 catenin (1:1000 dilution; BD), β-tubulin (1:100 dilution; Abcam, Cambridge, UK), flotillin (1:500 dilution; BD), rabbit polyclonal antibodies to tyrosine phosphorylated p120 (phospho Y228) (1:500; Abcam), GFP (1:500 dilution, Medical Biological Laboratory, Nagoya, Japan). Secondary antibodies included Cy3-labeled antibodies to mouse or rabbit immunoglobulin G (1:1000 dilution; Jackson ImmunoReseach Laboratory) and Alexa Fluor 488-labeled antibodies to mouse or rabbit immunoglobulin G (1:1000 dilution; Jackson ImmunoReseach Laboratory). Cell nuclei were stained with 2,4-diamidino-2-phenylindole dihydrochloride *n*-hydrate (DAPI) (0.5 μg ml^-1^, Sigma). Samples were visualized using an inverted microscope (model IX71; Olympus, Tokyo, Japan) and a CCD camera (DP70; Olympus). Images were post-processed using Adobe Photoshop (Adobe Systems, San Jose, CA).

### Immunoblotting and immunoprecipitation

Sample preparation and immunoblot analyses were performed as described [21,22,24]. Immune complexes were detected by colorimetry with a BCIP/NBT detection kit (Sigma). Immunoprecipitation was done with a Pierce Crosslink Magnetic IP/Co-IP Kit (Thermo Fisher Scientific Inc., Rockford, IL). Threonine phosphorylation of p120 was detected by rabbit polyclonal antibodies to threonine phosphorylated p120 (phospho T310) (1:500 dilution; Abcam).

### Time-lapse recording

Cells were cultured in neutral red-depleted pmDM and placed in a humid chamber (Tokai Hit, Fujinomiya, Japan) maintained at 37°C under 10% CO_2_. Time-lapse images were taken using an inverted microscope (BZ9000; Keyence, Osaka, Japan) with a 10× or 20× Plan Apo Fluor objective lens (Nikon, Tokyo, Japan).

### Quantification of muscle cell hypertrophy

The distribution of myogenic cell sizes was determined by calculating the percentage of nuclei in myogenic cells with different numbers of nuclei in the total number of nuclei (myoblasts plus myotubes), as described previously [5].

### Fractionation of detergent-resistant membranes

Ric10 cells were cultured for 24 h in pmGM and then further incubated for 24 h in pmDM. The cells were lysed in 0.2 ml of ice-cold lysis buffer (0.5% Trion X-100, 50 mM MES (pH 6.0), 50 mM NaCl, 5 mM MgCl_2_, and 2.5 mM EGTA) containing protease inhibitor (Complete Protease Inhibitor Cocktail EDTA-free; Roche, Mannheim, Germany) for 30 min on ice. Protein concentrations in aliquots of cell lysates were determined using a BCA kit (Sigma). An aliquot of the lysate containing approximately 300–400 μg protein was mixed with OptiPrep (Axis-Shield, London, UK) and fractionated in a 3 ml Optiprep gradient according to the manufacturer’s instructions (Caveolae/Rafts Isolation Kit; Sigma). Ten fractions were collected from the top, and 30 μl of each fraction was analyzed by immunoblotting. The PVDF membranes were scanned, and the signal intensity of each band was quantified using Image J software (NIH). Detergent-resistant membrane (DRM) fractions consist of lipid rafts. The distribution of the protein in each fraction was determined by calculating the ratio of the signal intensity of the protein band in each fraction to the sum of the signal intensity in all fractions. In the indicated experiments, the amounts of M-cadherin and p120 in lipid rafts were quantified using Flotillin-1 as a standard. To detect the raft-specific ganglioside GM1 in DRM, 30 μl of each fraction was spotted on a nitrocellulose membrane, probed with HRP-conjugated cholera toxin B subunit (CTB), then detected by colorimetry using a Fast DAB kit (Sigma). The distribution of GM1 in each fraction was determined by calculating the ratio of the signal intensity of the spot in each fraction to the sum of the signal intensity in all fractions.

## Results

### M-cadherin is recruited to lipid rafts during myogenic differentiation

**Figure 1:**
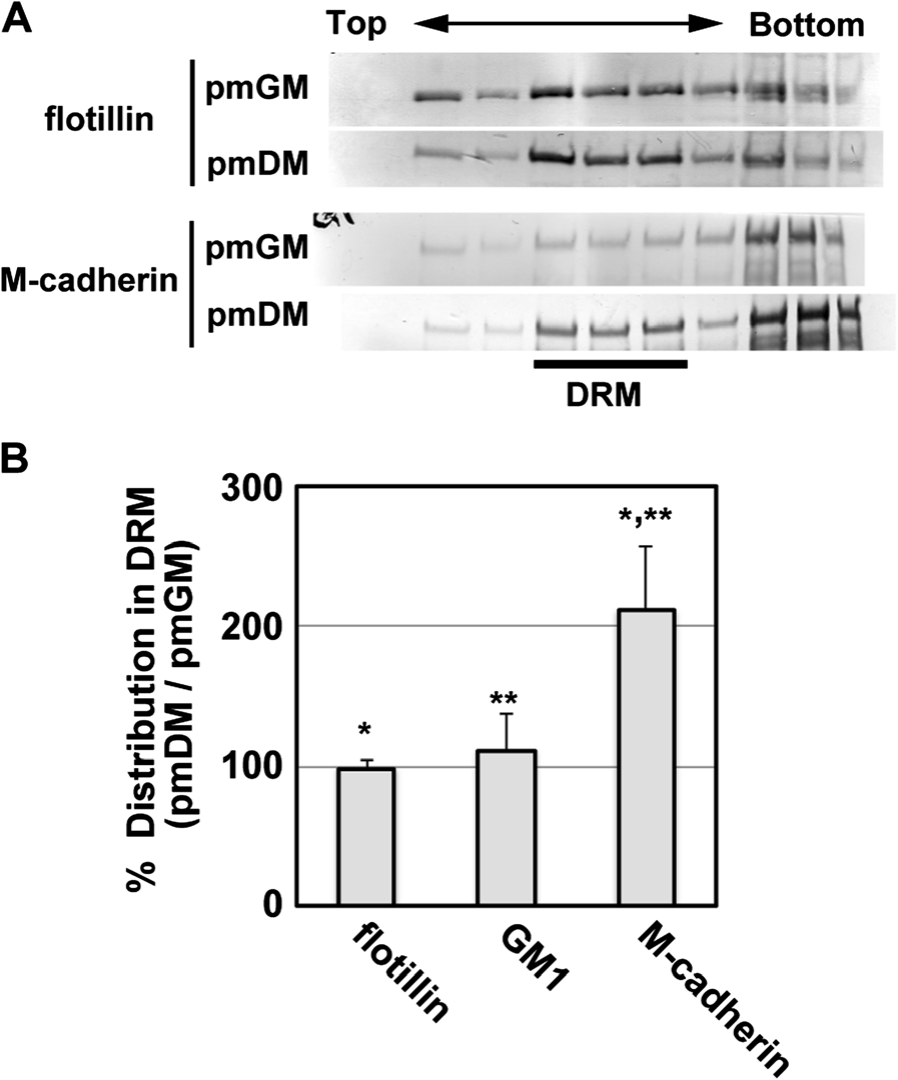
**M-cadherin and p120 catenin form protein complex at lipid raft during myogenic differentiation.** GGS25 cells were cultured in pmDM for 24 h at low cell density to avoid cell-cell contact. Cells were subjected to fractionation of the detergent-resistant membrane (DRM) fractions. **(A)** The distribution of M-cadherin in each fraction (30 μl) of growing (pmGM) or differentiating (pmDM) GGS25 cells was analyzed by immunoblotting with M-cadherin antibody. The line represents the detergent-resistant membrane fractions. **(B)** The distributions of M-cadherin, flotillin, and GM-1 in DRM fractions of differentiating cells are represented as the % of the amount in DRM fraction of growing cells. M-cadherin and flotillin were analyzed by immunoblotting using the appropriate antibodies. GM-1 was analyzed by dot blot assay with HRP-labeled CTB. Flotillin and GM-1 were used as markers of lipid rafts. Averages and standard deviations (n = 3) are shown and analyzed using Student’s *t*-test. *P < 0.1, **P < 0.05.

Dynamic clustering and dispersion of lipid rafts at the leading edge of lamellipodia and membrane ruffles that contain presumptive fusion sites is critical for myogenic cell fusion [10]. We focused on a molecular mechanism underlying recruitment of M-cadherin to the presumptive fusion site. GGS25 [10] is a mouse myogenic cell line that constitutively expresses GPI-anchored GFP (GFP-GPI) as a raft marker. Cells were cultured at low cell density to avoid cell-cell contact. Subcellular fractionation of GGS25 cells by density gradient ultracentrifugation showed that most M-cadherin was located at non-raft membranes in growing myogenic cells (Figure 1A). M-cadherin was recruited to lipid rafts under the terminal muscle differentiation-inducing condition, whereas the concentrations of raft marker molecules, flotillin and ganglioside GM1, remained constant during myogenesis (Figure 1A,B). The results were consistent with the previous observation showing that M-cadherin is accumulated at lipid rafts in cell contact-free regions of plasma membrane [10].

### Active Rac1 enhances myogenic cell fusion

The small G protein Rac1 is known to play a pivotal role in lamellipodium formation in various cell types. Rac1 physically interacts with M-cadherin [25] and is activated by p120 [26,27] and M-cadherin [25]. Therefore, we determined whether Rac1 is involved in the recruitment of M-cadherin to lipid rafts during myogenic cell fusion.

**Figure 2:**
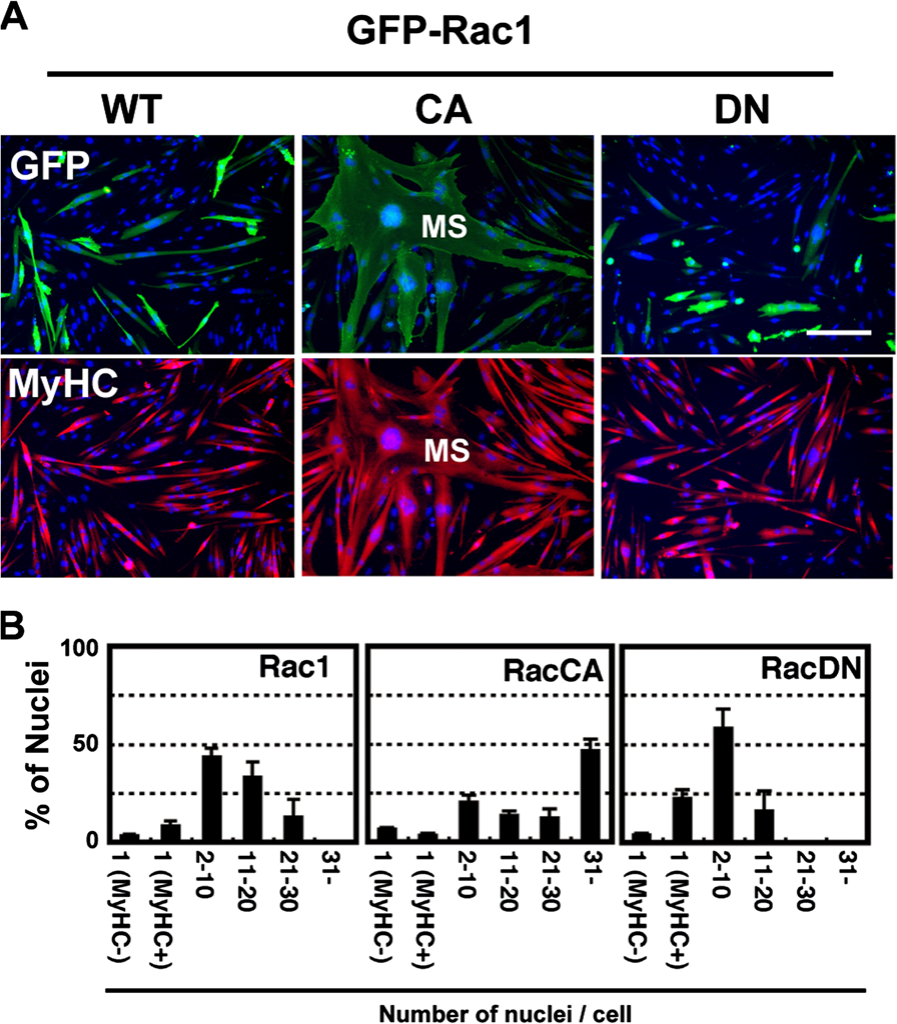
**Rac1 enhances myogenic cell fusion.** Expression plasmids encoding GFP-Rac1 (WT, wild type; CA, constitutively active form; DN, dominant negative form) were transfected into Ric10 cells under the differentiation-inducing condition. **(A)** The cells were cultured for 24 h, fixed and subjected to immunostaining for GFP (green) and MyHC (red). Cell nuclei were stained with DAPI (blue). Images were obtained by phase contrast and epifluorescence microscopy. MS, myosheet. Scale bar, 100 μm. **(B)** Histograms represent the distribution of myogenic cells with different numbers of nuclei in cells expressing GFP-Rac1. Mononucleated cells were classified to two subpopulations: one expressing MyHC (MyHC+) and the other not (MyHC-). Averages and standard deviations (n = 3) are shown.

Expression plasmids encoding GFP-fused wild (WT), constitutively active (CA), or dominant negative (DN) Rac1 were transfected into Ric10 cells under the differentiation-inducing condition to avoid the inhibitory effects of Rac1 on the initiation of myogenesis [28,29]. Therefore, MyHC was expressed in 97.0%, 94.0%, or 96.3% of Ric10 cells transfected with GFP-Rac1WT, CA, or DN expression plasmids, respectively (Figure 2A). Fusion indexes were 89.0%, 91.1%, or 73.6% in Ric10 cells expressing Rac1WT, CA, or DN, respectively. In addition, Rac1CA-expressing Ric10 cells gave rise to large multinucleated cells called myosheets [5] containing more than 31 nuclei (Figure 2 middle panels in A and B). It suggests that Rac1CA promotes cell fusion between myotubes or myoblasts and myotubes as well as myoblasts. In contrast, Ric10 cells expressing Rac1DN formed small myotubes exclusively (Figure 2 right-hand panels in A and B). The results indicated that Rac1 promotes myogenic cell fusion depending on its activity.

### Rac1 activity is required for microraft organization at presumptive fusion sites

**Figure 3:**
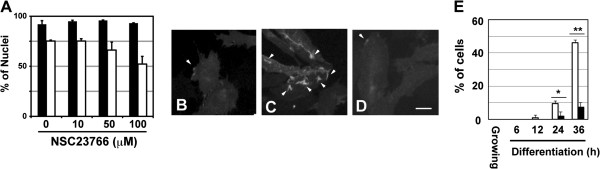
**Rac1 inhibitor suppresses microraft organization during myogenic differentiation. (A)** GGS25 cells were cultured for 24 h and then further cultured in pmDM supplemented with 0.1% DMSO or NSC23766 (10–100 μM) for up to 36 h. Differentiated cells were detected by immunostaining with anti-MyHC antibody (filled columns). Fusion indexes were calculated as percentages of nucleus numbers in multinucleated cells (open columns). Averages and standard deviations (n = 3) are shown. **(B**-**E)** GGS25 cells were cultured in pmGM **(B)**, pmDM with 0.1% DMSO (C and E), or pmDM with NSC23766 (100 μM) for up to 37 h **(D** and **E)**. GFP-GPI was sequentially observed at 1 min intervals under epifluorescence microscopy by time-lapse recording. Pictures were taken after 24 h of differentiation cultures **(C** and **D)** or growing culture **(B)**. Scale bar, 25 μm. **(E)** Percentage of cells that showed membrane ruffles during 1 h following the indicated time in pmDM supplemented with DMSO (open columns) or NSC23766 (filled columns). Averages and standard deviations (n = 3) are shown and analyzed using Student’s *t*-test. *,**P < 0.05.

GFP-Rac1DN prevented lamellipodium formation and often induced a severely abnormal morphology in Ric10 cells (data not shown), implying that it produced unphysiologically severe damage to the cells, perhaps due to the disordered arrangement of cytoskeletons. Therefore, we used the Rac1 inhibitor NSC23766 to inhibit Rac1 activity to more moderate levels. NSC23766 did not inhibit the expression of MyHC at 100 μM (Figure 3A), although it attenuated myogenesis of Ric10 at 200 μM (data not shown). However, 100 μM NSC23766 inhibited myogenic cell fusion without compromising the expression of MyHC (Figure 3A), as previously described in C2C12 cells [25].

To determine the role of Rac1 in microraft organization, GGS25 cells expressing GFP-GPI were treated with NSC23766. Lipid rafts were visualized in living cells by GFP-GPI under an epifluorescence microscope. Microrafts were rarely organized under the growing condition (Figure 3B and Additional file 1), but organization of microrafts was markedly promoted at membrane ruffles under the differentiation-inducing condition (Figure 3C and Additional file 2). Intensive GFP fluorescence at membrane ruffles of differentiating GGS25 cells showed dense clusters produced by a large number of nanometer-sized lipid rafts. In contrast, microraft organization was severely suppressed in differentiating GGS25 cells treated with NSC23766 (Figure 3D and Additional file 3).

To quantify the inhibitory effects of NSC23766 on microraft organization, a microraft visualized by GFP-GPI was observed by time-lapse recording for 1 h from the indicated time in Figure 3E. The number of cells that organized microrafts at membrane ruffles markedly increased under the differentiation-inducing condition (Figure 3C and E). The frequency of microraft organization at lamellipodia of an individual cell also increased (Additional files 1 and 2). In contrast, NSC23766 prevented microraft organization at membrane ruffles (Figure 3D and E). The frequency of microraft organization at lamellipodia severely declined in NSC23766-treated cells compared to that of untreated cells (Additional file 3). These results showed that Rac1 plays a pivotal role in microraft formation at the presumptive fusion site and suggest that organization of microraft is essential for myogenic cell fusion.

### Rac1 activity is dispensable for recruitment of M-cadherin to lipid rafts and interaction with p120 catenin

**Figure 4:**
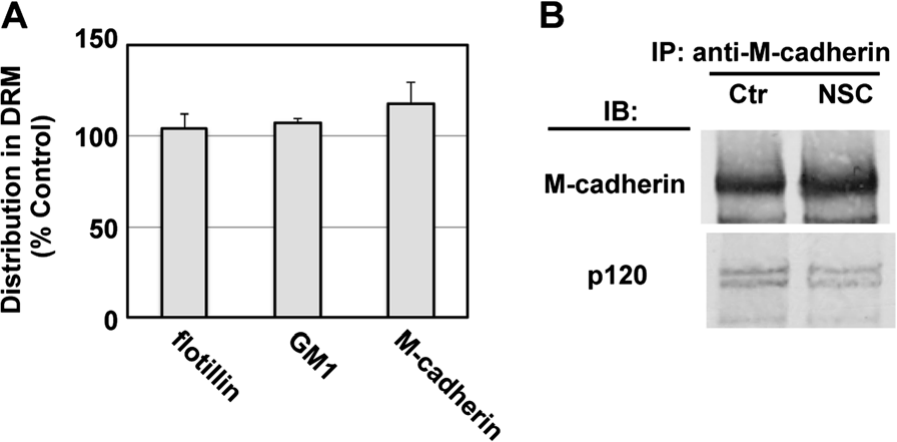
**Interaction between p120 and M-cadherin is independent of Rac1 activity.** Ric10 cells were cultured in pmGM for 24 h and then further cultured in pmDM for up to 24 h. **(A)** Plasma membrane was fractionated by density-gradient ultracentrifugation. DRM fractions were pooled and subjected to immunoblot analysis for flotillin and M-cadherin and dot blot analysis for GM-1. The distribution of molecules in DRM fractions of NSC23766-treated cells is represented as the % of the amount in DRM fraction of untreated control cells. Averages and standard deviations (n = 3) are shown. **(B)** Ric10 cells were cultured for 24 h in pmDM supplemented with 0.1% DMSO (Ctrl) or 100 μM NSC23766 (NSC). Then, immunoprecipitated materials from total cell lysates were subjected to immunoblot analysis. Similar results were obtained by two independent experiments. Representative results were shown.

In the next series of experiments, we determined whether a Rac1-dependent organization of microrafts is required for differentiation-induced recruitment of M-cadherin to lipid rafts. The plasma membrane was fractionated by density gradient ultracentrifugation. NSC23766 did not affect the amounts of M-cadherin and raft markers flotillin and GM1 in the raft fraction (Figure 4A). Furthermore, similar concentrations of p120 were found in immunoprecipitates from both control and NSC23766-treated Ric10 cells with anti-M-cadherin antibody (Figure 4B). Thus, microraft organization might be regulated independently of the recruitment of M-cadherin and p120 to nanometer-sized lipid rafts and their physical interaction.

### M-cadherin interacts with p120 catenin predominantly at lipid rafts during myogenic differentiation

**Figure 5:**
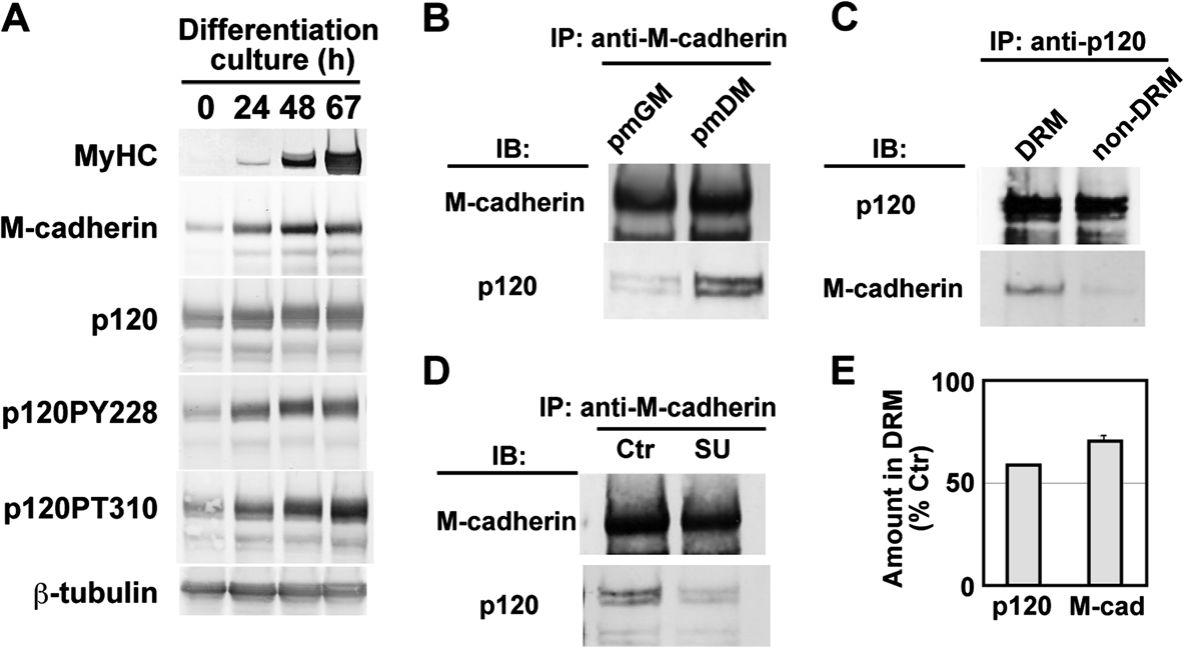
**Src kinase inhibitor impairs physical interactions between M-cadherin and p120 catenin and their recruitment to lipid rafts. (A)** Total lysates (20 μg of proteins) were prepared from Ric10 cells cultured in pmDM for 0 h (lane 1), 24 h (lane 2), 48 h (lane 3), or 67 h (lane 4), and then subjected to immunoblot analysis for the represented proteins. MyHC, myosin heavy chain; p120PY228, tyrosine phosphorylated p120; p120PT310, threonine phosphorylated p120. β-tubulin was used as a loading control. **(B)** M-cadherin of growing or differentiating Ric10 cells was immunoprecipitated and analyzed by immunoblotting with the appropriate antibodies. **(C)** p120 catenin was immnoprecipitated from the DRM or non-DRM fractions of differentiating Ric10 cells, and then analyzed by immunoblotting with the appropriate antibodies. **(D)** M-cadherin was immunoprecipitated from total cell lysates of untreated (Ctrl) or SU6656-treated Ric10 cells (SU), and then analyzed by immunoblotting with the appropriate antibodies. **(E)** The distribution of M-cadherin and p120 catenin in DRM fractions (30 μl) of untreated or SU6656-treated Ric10 cells was analyzed by immunoblotting with the appropriate antibodies. The distribution of M-cadherin and p120 catenin in DRM was estimated as the relative amount normalized by the amount of flotillin in DRM fractions. Then the distribution of M-cadherin and p120 in DRM fractions of SU6656-treated cells was represented as the % of the amount in DRM fraction of untreated control cells. Averages and standard deviations (n = 3) are shown.

Cell contact-independent recruitment of adhesion molecules to lipid rafts is likely to be a critical step of myogenic cell fusion [10]. P120 catenin is a probable candidate for a key molecule in recruitment of M-cadherin because it regulates the locations of N-cadherin in lipid rafts [30]. The amounts of M-cadherin increased prior to the expression of the muscle-differentiation marker myosin heavy chain (MyHC) (Figure 5A). The amounts of p120 catenin increased slightly. In addition, a specific antibody against the tyrosine phosphorylated form of p120 revealed that its phosphorylation was robustly enhanced during myogenic differentiation (Figure 5A). Phosphorylation of threonine residues of p120 was also enhanced. Co-immunoprecipitation assays indicated that physical interaction between M-cadherin and p120 was promoted during myogenic differentiation (Figure 5B). In addition, the complex including M-cadherin and p120 catenin was found predominantly in lipid rafts (Figure 5C). The results suggest that M-cadherin is associated with p120 in a lipid raft–dependent fashion during myogenic differentiation.

To determine the role of Src family kinases in interaction of p120 with M-cadherin, Ric10 cells were treated with the Src kinase inhibitor SU6656 because Src family kinases are major protein kinases that phosphorylate tyrosine residues of p120 [31]. SU6656 markedly attenuated the interaction between p120 and M-cadherin (Figure 5D). Furthermore, the amounts of M-cadherin and p120 at lipid rafts declined in SU6656-treated cells (Figure 5E). Recruitment of N-cadherin to lipid rafts was also attenuated by SU6656 in a manner similar to M-cadherin (data not shown). SU6656 attenuated physical interaction between M-cadherin and p120 catenin, and their recruitment to lipid rafts, implicating the role of Src family kinases in the regulation of recruitment of M-cadherin to lipid rafts.

### Src kinase inhibitor suppresses myogenic cell fusion without compromising myogenic differentiation

**Figure 6:**
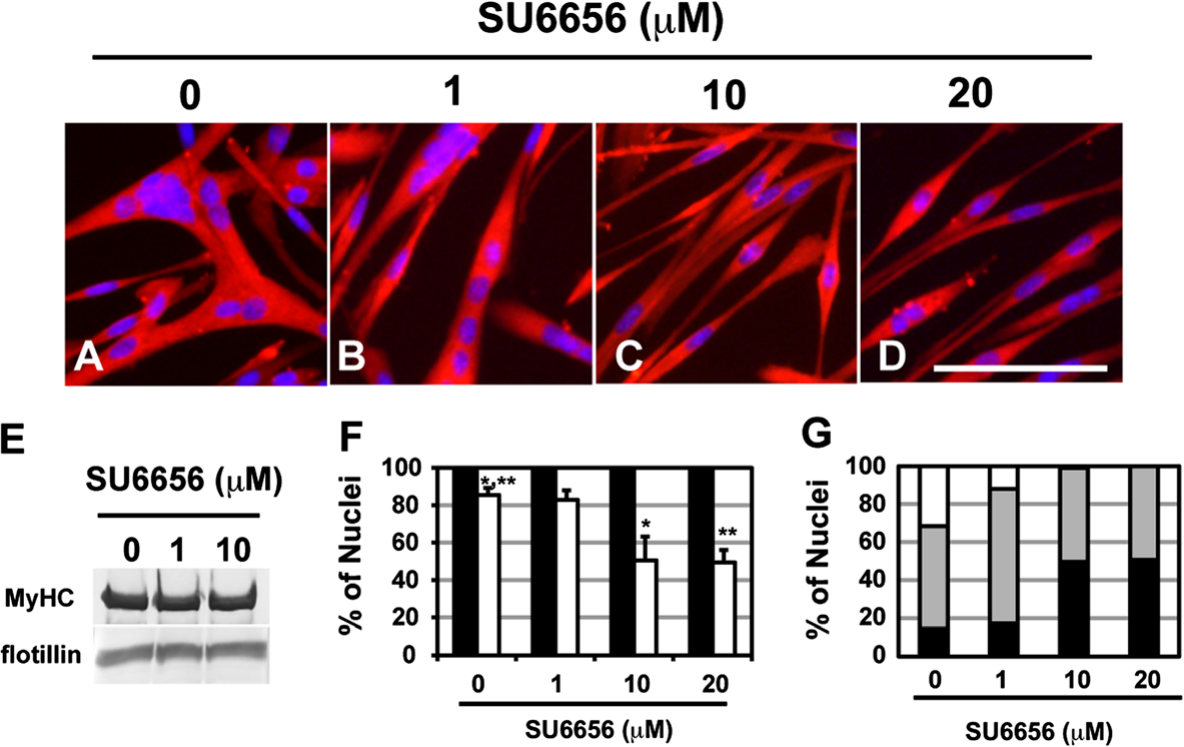
**Src kinase inhibitor prevents myogenic cell fusion.** Ric10 cells were cultured for 24 h in pmDM and then further cultured in pmDM supplemented with 0.1% DMSO or SU6656 for further 24 h. **(A-D)** The cells were fixed and subjected to immunostaining for MyHC (red). Cell nuclei were stained with DAPI (blue). Images were obtained by epifluorescence microscopy. Scale bar: 100 μm. **(E)** Total cell lysates (20 μg) were subjected to immunoblot analysis for the represented proteins. Flotillin, a marker of lipid rafts, was used as a loading control. **(F)** Differentiated cells were detected by immunostaining with anti-MyHC antibody (filled columns). Fusion indexes were calculated as percentages of nuclear numbers in multinucleated cells (open columns). Averages and standard deviations (n = 7) are shown and analyzed using Student’s *t*-test. *P < 0.002, **P < 0.0002. **(G)** Histograms represent the distribution of myogenic cells with different numbers of nuclei in unstimulated and SU6656-stimulated cultures. The cells were classified to four subpopulations: myotubes containing more than 11 nuclei (white columns), myotubes containing 2–10 nuclei (grey columns), mononucleated cells expressing MyHC (black columns), and mononucleated cells not expressing MyHC (less than 1% in the experiments).

Recruitment of M-cadherin to lipid rafts is supposed to be indispensable for its accumulation at the presumptive fusion site [10]. Actually, SU6656 suppressed myogenic cell fusion in a dose-dependent manner (Figure 6A-D, F and G), although it did not inhibit expression of the myogenic differentiation marker MyHC (Figure 6A-F), M-cadherin, or p120 (Additional file 4A). Inhibition of cell fusion by SU6656 resulted in a severe reduction in the number of larger myofibers containing more than 11 nuclei (Figure 6G).

### Src kinase inhibitor suppresses accumulation of tyrosine-phosphorylated p120 and organization of microrafts

Despite the robust inhibition by SU6656 of recruitment of M-cadherin to lipid rafts and myogenic cell fusion, antibodies recognizing phosphorylated tyrosine residue 228 of p120 did not show a decline in the amounts of tyrosine phosphorylated p120 in the total cell lysate (Additional file 4A). In addition, the amounts of tyrosine phosphorylated p120 increased when treated with the tyrosine phosphatase inhibitor vanadate (Additional file 4B). Thus, turnover of phosphorylated tyrosine residues of p120 might depend predominantly on tyrosine phosphatase. The results suggest that other Src kinase substrates are involved in physical interaction between M-cadherin and p120 catenin, and their recruitment to lipid rafts.

**Figure 7:**
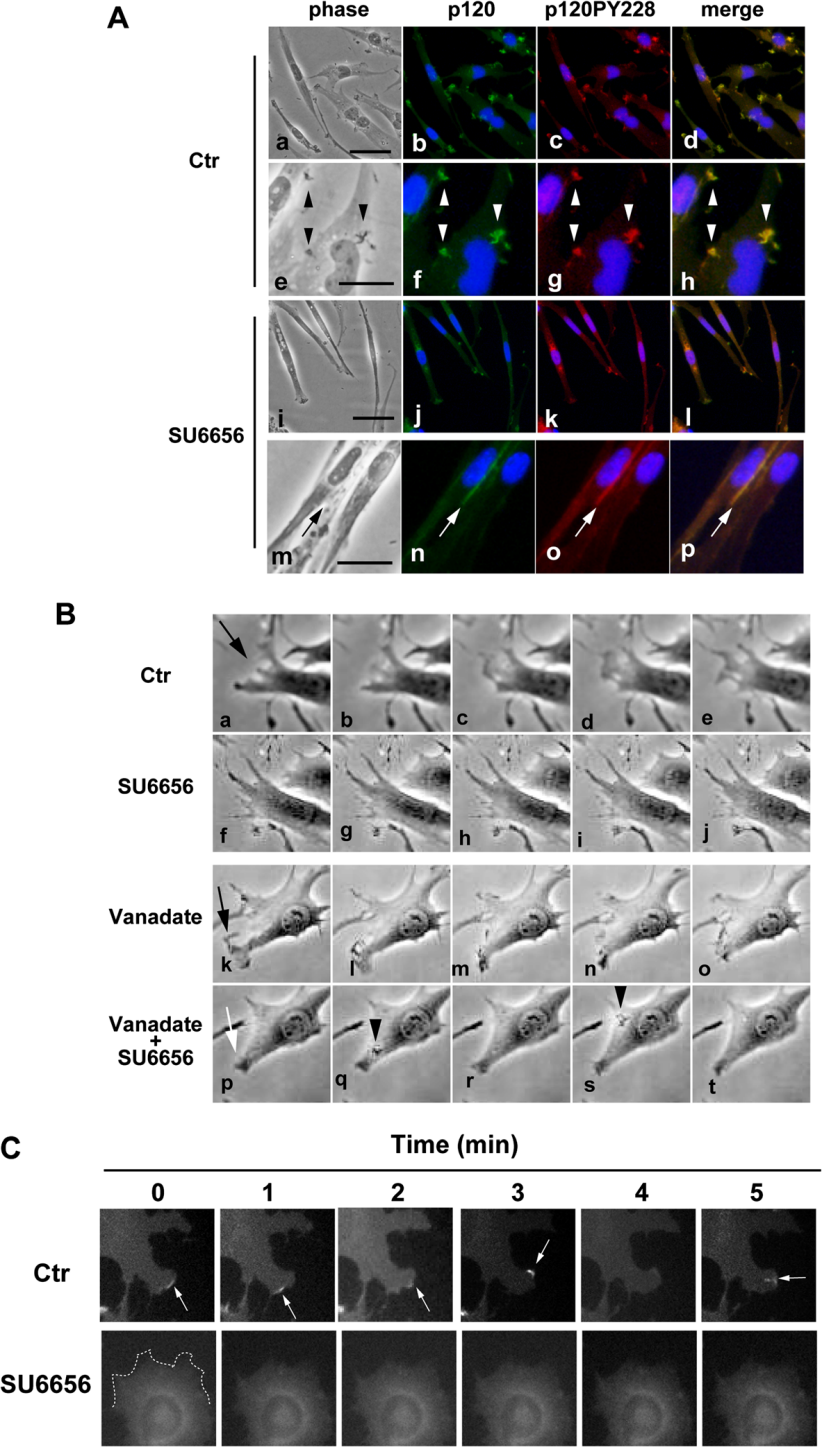
**Src kinase inhibitor suppresses accumulation of p120 and organization of microrafts. (A)** Ric10 cells were cultured for 24 h in pmDM and then further cultured in pmDM supplemented with 0.1% DMSO (a-h) or SU6656 (i-p) for further 9 h. The cells were fixed and subjected to immunostaining for p120 (green) and p120PY228 (red). Cell nuclei were stained with DAPI (blue). Images were obtained by phase contrast and epifluorescence microscopy. Arrowheads represent membrane ruffles (e-h). Arrows represent cell contacts (m-p). Scale bars: 100 μm (a-d, i-l), 25 μm (e-h, m-p). **(B)** Ric10 cells were cultured in pmDM for 24 h and sequentially observed under phase contrast microscopy by time-lapse recording with 2.5 min interval (upper row). Then, the medium was switched to pmDM supplemented with SU6656 (10 μM). The same field was sequentially observed approximately 10 minutes after administration (second row). Ric10 cells were cultured for 24 h in pmDM and then incubated for 20 min in pmDM supplemented with 100 μM vanadate. The cells were sequentially observed by time-lapse recording (third row). Then the medium was switched to pmDM supplemented with both 100 μM vanadate and 10 μM SU6656 and sequentially observed by time-lapse recording (lowest row). Arrows represent the leading edge of lamellipodium. Arrowheads represent membrane ruffles. **(C)** GGS25 cells were cultured for 24 h in pmDM and then further cultured for 3 h in pmDM supplemented with 0.1% DMSO (upper row) or 10 μM SU6656 (lower row). The cells were sequentially observed at 1 min intervals under epifluorescence microscopy by time-lapse recording. Arrows represent microrafts where GFP-GPI was accumulated.

It is likely that subcellular localization of p120 is critical to myogenic cell fusion. Therefore, the effects of SU6656 on the subcellular distribution of tyrosine-phosphorylated p120 in differentiating myogenic cells were determined. Immunostaining analyses showed that tyrosine phosphorylated p120 accumulated at membrane ruffles (Figure 7Aa-h). SU6656 inhibited the distribution of p120 at membrane ruffles (Figure 7Ai-l). Thus, Src family kinases might induce the redistribution of p120 at membrane ruffles through phosphorylation of other substrates. In contrast, p120 accumulated at cell contacts even in SU6656-treated cells (Figure 7Am-p) as well as in control cultures (Additional file 5).

To determine whether SU6656 inhibits membrane ruffling in differentiating myogenic cells, Ric10 cells were sequentially observed by time-lapse recording. The plasma membrane of myogenic cells frequently ruffled during myogenic differentiation (Figure 7B top row and Additional file 6). SU6656 prevented membrane ruffling within 10 minutes after administration (Figure 7B second row and Additional file 7). The tyrosine phosphatase inhibitor vanadate did not affect membrane ruffling of Ric10 cells (Figure 7B third row and Additional file 8). However, a 20-min pretreatment with a high concentration of vanadate (100 μM) partially but clearly antagonized the inhibitory effect of SU6656 on membrane ruffling of Ric10 cells (Figure 7B bottom row and Additional file 9). The results suggest that tyrosine phosphorylation under the control of Src kinase is involved in the regulation of membrane ruffling.

To obtain direct evidence that SU6656 affects the organization of microrafts, GFP-GPI in GGS25 cells was sequentially observed by time-lapse recording. Rapid cycles of clustering and dispersion of microrafts were visualized by GFP-GPI under epifluorescence microscopy (Figure 7C upper panels and Additional file 10). In contrast, SU6656 perfectly suppressed the organization of microrafts (Figure 7C lower panels and Additional file 11).

Taken together with the results above, it is suggested that Src kinase plays a critical role in recruitment of M-cadherin/p120 to lipid rafts. In addition, the results imply that the recruitment of adhesion proteins to lipid rafts might be relevant to microraft organization at the presumptive fusion site.

## Discussion

Dynamic clustering and dispersion of lipid rafts is required to establish fusion-competent sites on the myogenic cell membrane [10]. Although cell adhesion and membrane fusion are sequential steps in myogenic cell fusion, both steps require distinct microcircumstances in the plasma membrane. To establish cell adhesion, the plasma membrane at the presumptive fusion site must contain enough cholesterol to maintain the rigid lipid bilayers that hold adhesion complexes. However, membrane fusion takes place at cholesterol-free spots of the plasma membrane [11,32,33], and adhesion complexes are removed from the fusion site prior to membrane breakdown/union [10]. The dynamic clustering and dispersion of lipid rafts enables robust and rapid changes in plasma membrane components at the presumptive fusion site. However, the pre-fusion events required for specialization of fusion-competent sites have been unknown. The present study showed that both the recruitment of adhesion complexes to lipid rafts and the organization of microrafts are required for establishing fusion-competent sites.

One possible mechanism of M-cadherin recruitment to lipid rafts could be based on control of its binding with p120 catenin. P120 is essential for stability of cadherins on the plasma membrane in a direct interaction-dependent manner [34,35]. Tyrosine-phosphorylated p120 shows increased affinity to cadherins [36-39]. Tyrosine phosphorylation of p120 also requires recruitment of p120 to plasma membranes [31,40,41]. In addition, only membrane-associated Src can phosphorylate p120 [42]. However, we were unable to detect a significant difference in the total amounts of tyrosine-phosphorylated p120 between control and Src kinase inhibitor SU6656-treated differentiating myogenic cells. A major fraction of tyrosine-phosphorylated p120 retained the phosphorylated state even when Src family kinases were inhibited. Despite that, SU6656 inhibited differentiation-induced recruitment of M-cadherin/p120 to lipid rafts and their physical interaction. Thus, in the physiological cellular context, another Src kinase substrate might play a role in the recruitment of M-cadherin/p120 to lipid rafts. Actually, Src kinase stimulates the E-cadherin regulator protein to regulate cell-cell adhesion [43]. Crosstalk between cAMP-dependent protein kinase (PKA) and Src pathways [44,45] is another possible mode of Src kinase action for M-cadherin recruitment because the localized PKA pathway is involved in the specialization of the fusion-competent areas of the plasma membrane in myogenic cells [5].

SU6656 prevented both microraft organization and membrane ruffling. Src family kinases bind to the autophosphorylated focal adhesion kinase (FAK) that is activated by integrin-mediated adhesion [46]. The active FAK-Src complex stimulates Rac1 activity through phosphorylation of a number of mediators including the scaffolding protein p130Cas, paxillin, paxillin kinase linker, Pak-interacting exchange factor-beta, and spleen tyrosine kinase. Thus, Src is likely to play a role in microraft organization through stimulation of Rac1 activity.

P120 catenin also modulates the activity and spatial distribution of Rac1 [25,47-49]. Either SU6656 or the Rac1 inhibitor NSC23766 inhibited the organization of microrafts, the generation of membrane ruffles/lamellipodia, and cell fusion of myogenic cells under the differentiation-inducing condition. We were unable to detect a significant difference between control and SU6656-treated cells in the total Rac1 activity by a pull-down assay. However, we did see effects of SU6656 on differentiating myogenic cells similar to those of NSC27366. The p120-knockdown inhibits the lamellipodia dynamics and localization of Rac1 but doesn’t decrease the total Rac1 activity [49]. SU6656 might modulate the spatial distribution of Rac1, as shown in p120 knockdown cells.

**Figure 8:**
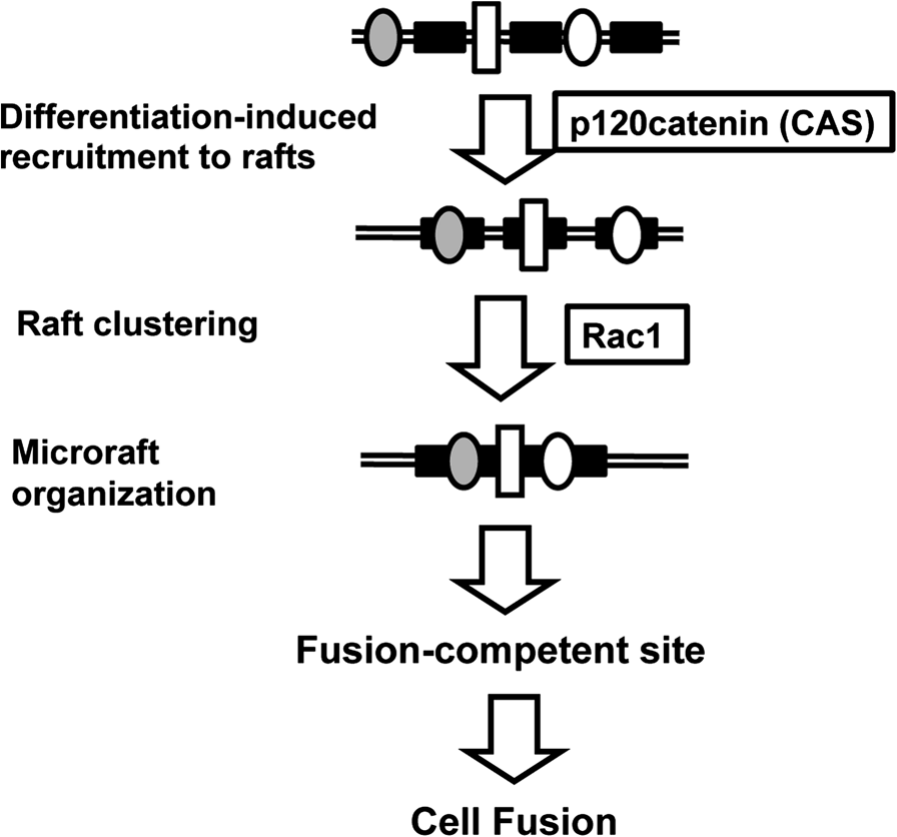
**Procedure establishing fusion-competent sites consists of two sequential events: the recruitment of adhesion complexes to lipid rafts and the organization of microrafts.** The recruitment of cadherins to lipid rafts depends on the interaction with p120 catenin, whereas the organization of microrafts is controlled by a small G protein, Rac1. Filled squares, lipid rafts; circles and open squares, adhesion proteins.

Subcellular distribution of cadherins is determined by at least two distinct dynamic cycles: the *trans*-directional membrane-cytoplasmic transport/endocytosis cycle [50] and the *cis*-directional lateral clustering/dispersion cycle within the plasma membrane [10,51,52]. The underlying mechanism controlling the recruitment of cadherin to lipid rafts remained to be discerned, while relevant factors, including clathrin and p120 catenin, have been proposed for regulation of the membrane transport/endocytosis cycle [53,54]. In the *cis*-cycle, the distribution of M-cadherin in lipid rafts or non-raft regions of the plasma membrane corresponds to a dynamic equilibrium of association and dissociation with p120. The accumulation of M-cadherin at microrafts is found at both cell contact and presumptive fusion sites of myogenic cells [10]. The present study shows that M-cadherin is recruited to lipid rafts independently of the microraft organization. The previous and present studies imply that the functional M-cadherin/p120 catenin complex is first recruited to nanometer-sized lipid rafts (nanorafts). Then, either cell-cell contact or myogenic differentiation induces nanorafts to give rise to a microraft, resulting in the robust accumulation of cadherin/p120 complexes at the cell contact and presumptive fusion site (Figure 8).

N-cadherin as well as M-cadherin plays a role in myogenic cell fusion [55]. N-cadherin also accumulates at microrafts at ruffling membranes and the leading edge of lamellipodia of differentiating myogenic cells [10]. P120 is involved in the regulation of N-cadherin location in lipid rafts [30]. In addition, the present study shows that SU6656 also suppressed the recruitment of N-cadherin to lipid rafts. Taken together, the recruitment of N-cadherin and M-cadherin to lipid rafts might have common mechanisms in differentiating myogenic cells.

Cadherins are involved in cell recognition and adhesion by homophilic interactions conferred by their extracellular regions. Their intracellular regions link them with cytoplasmic partner proteins and consequently the actin filament network. Thus, our findings on the accumulation of cadherins at the presumptive fusion sites might be related to remodeling of the actin cytoskeleton. We previously analyzed the arrangement of F-actin during myogenic cell fusion [5,10]. Briefly, cadherin complex is co-aligned with F-actin at ruffling membranes and the leading edge of lamellipodia. The cortical actin cytoskeleton at presumptive fusion sites might play a critical role in lateral dispersion of lipid rafts. Interestingly, after myogenic cell fusion, both the ruffling membrane and lamellipodium disappear except at the polar ends of myotubes [5]. The localized accumulation of cadherins at the polar ends of myotubes might create anchoring points of actin filaments and contribute to remodeling actin cytoskeleton during myogenic differentiation.

The present results suggest that the recruitment of M-cadherin/p120 complex to lipid rafts of cell contact − free surfaces is essential for the specialization of fusion-competent areas of the plasma membrane. Src kinase activity is likely to be critical to the recruitment of M-cadherin to lipid rafts. Rac1 induces the dynamic organization of microrafts at the presumptive fusion site. However, the mechanism connecting these two sequential events has not been discerned. It is likely that Rac1 is activated downstream of Src kinase and M-cadherin/p120 during myogenic differentiation [25,46-48]. The present study provides a possible molecular mechanism underlying the specialization of presumptive fusion sites of myogenic cells.

## Conclusions

Myoblast fusion consists of a series of steps including plasma membrane breakdown/union that is initially induced in a discrete area of the plasma membrane.

However, the pre-fusion events that are relevant to specialization of fusion-competent sites of the plasma membrane remained to be discerned. Here we showed that the procedure establishing fusion-competent sites consists of two sequential events: the recruitment of adhesion complexes to lipid rafts and the organization of microrafts (Figure 8). The recruitment of M-cadherin to lipid rafts depended on interaction with p120 catenin, whereas the organization of microrafts was controlled by a small G protein, Rac1.

## Availability of supporting data

The data sets supporting the results of this article are included within the article and its additional files.

## Competing interests

The authors declare no competing interests.

## Authors’ contributions

AM and NH designed and performed experiments, provided critical reagents and tools, and wrote the manuscript. Both authors read and approved the final manuscript.

## Supplementary Material

Additional file 1 **Rac1 inhibitor suppresses microraft organization during myogenic differentiation.** GGS25 cells were cultured in pmGM. GFP-GPI was sequentially observed at 1 min intervals under epifluorescence microscopy by time-lapse recording. Many microrafts were organized in pmDM, whereas the frequency of microraft organization and the signal intensity of GFP-GPI declined in pmGM and pmDM with NSC23766. Click here for file

Additional file 2 **GGS25 cells were cultured in pmDM for up to 37 h.** GFP-GPI was sequentially observed at 1 min intervals under epifluorescence microscopy by time-lapse recording. Many microrafts were organized in pmDM. Click here for file

Additional file 3 **GGS25 cells were cultured in pmDM with NSC23766 (100 μM) for up to 37 h.** GFP-GPI was sequentially observed at 1 min intervals under epifluorescence microscopy by time-lapse recording. The frequency of microraft organization and the signal intensity of GFP-GPI declined in pmDM with NSC23766. Click here for file

Additional file 4 **Effects of Src kinase inhibitor or protein tyrosine phosphatase inhibitor vanadate on fusion–related proteins of myogenic cells.** Ric10 cells were cultured for 24 h in pmDM and then cultured in pmDM supplemented with 0.1% DMSO (−) or SU6656 (+) for 9 h (A), or vanadate (1, 10, 100 μM) for 24 h (B). Total lysates (20 μg of proteins) were subjected to immunoblot analyses. MyHC, myosin heavy chain; p120PY228, tyrosine phosphorylated p120; p120PT310, threonine phosphorylated p120. β-tubulin was used as a loading control. Click here for file

Additional file 5 **Src kinase inhibitor doesn’t suppress accumulation of p120 at cell contacts.** Ric10 cells were cultured for 24 h in pmDM and then cultured in pmDM supplemented with 0.1% DMSO (Ctrl) or SU6656 (100 μM) for 24 h. Tyrosine-phosphorylated p120 accumulated at cell-cell contacts in both control cultures (Ctrl) and SU6656- (green) and p120PY228 (red)-treated cultures. Cell nuclei were stained with DAPI (blue). Images were obtained by epifluorescence microscopy. Click here for file

Additional file 6 **Vanadate antagonizes the inhibitory effect of Src kinase inhibitor on membrane ruffling.** Ric10 cells were cultured in pmDM for 24 h and sequentially observed under phase contrast microscopy by time-lapse recording.Images were recorded every 2.5 min by phase-contrast time-lapse microscopy. Membrane ruffling in pmDM (Additional file 6) was suppressed in pmDM supplemented with SU6656 (Additional file 7). Membrane ruffling in pmDM supplemented with vanadate (Additional file 8) was not suppressed in pmDM supplemented with SU6656 and vanadate (Additional file 9). Click here for file

Additional file 7 **Ric10 cells were cultured in pmDM for 24 h and sequentially observed under phase contrast microscopy by time-lapse recording (Additional file****6****).** Then, the medium was switched to pmDM supplemented with SU6656 (10 μM). The same field was sequentially observed approximately 10 minutes after administration. Images were recorded every 2.5 min by phase-contrast time-lapse microscopy. Membrane rufflingwas suppressed in pmDM supplemented with SU6656. Click here for file

Additional file 8 **Ric10 cells were cultured for 24 h in pmDM and then incubated for 20 min in pmDM supplemented with 100 μM vanadate.** The cells were sequentially observed by time-lapse recording. Images were recorded every 2.5 min by phase-contrast time-lapse microscopy. Membrane ruffling in pmDM supplemented with. Click here for file

Additional file 9 **Ric10 cells were cultured for 24 h in pmDM and then incubated for 20 min in pmDM supplemented with 100 μM vanadate.** The cells were sequentially observed by time-lapse recording (Additional file 8). Then the medium was switched to pmDM supplemented with both 100 μM vanadate and 10 μM SU6656 and sequentially observed by time-lapse recording. Images were recorded every 2.5 min by phase-contrast time-lapse microscopy. Membrane ruffling in pmDM supplemented with vanadate (Additional file 8) was not suppressed in pmDM supplemented with SU6656 and vanadate. Click here for file

Additional file 10 **Src kinase inhibitor suppresses organization of microrafts.** GGS25 cells were cultured for 24 h in pmDM and then further cultured for 3 h in pmDM supplemented with 0.1% DMSO or 10 μM SU6656. Microrafts appeared as white spots and disappeared in control cultures (Additional file 10), whereas SU6656 prevented microraft organization and any plasma membrane movement (Additional file 11). Nothing moved in the latter file. Click here for file

Additional file 11 **GGS25 cells were cultured for 24 h in pmDM and then further cultured for 3 h in pmDM supplemented with 10 μM SU6656.** Images were recorded every min for 20 minutes by epifluorescence time-lapse microscopy. SU6656 prevented microraft organization and any plasma membrane movement. Nothing moved in the present movie. Click here for file
