# Supplementary figures and images for: Regulation of pre-fusion events: recruitment of M-cadherin to microrafts organized at fusion-competent sites of myogenic cells
Source: BMC Cell Biol. 2013 Aug 27;14:37. doi: 10.1186/1471-2121-14-37 (PMC3846853; doi:10.1186/1471-2121-14-37)

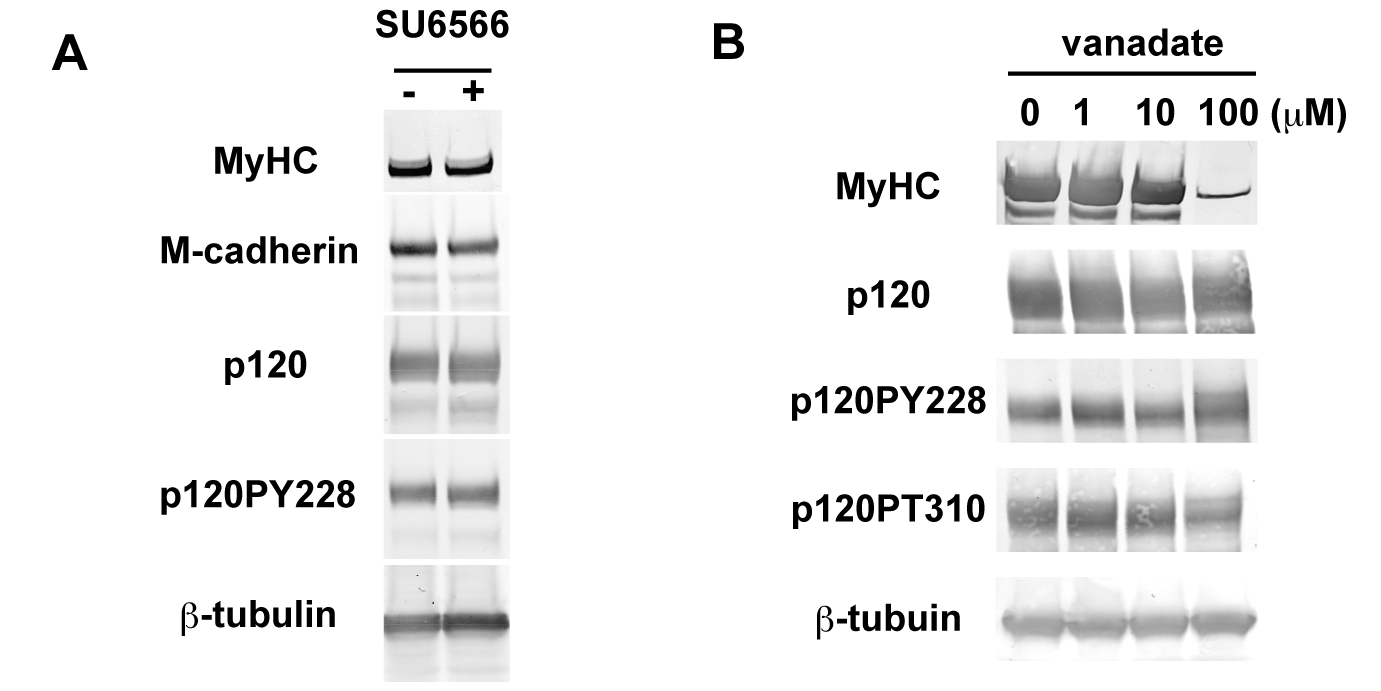

Supplement: Additional file 4 — Effects of Src kinase inhibitor or protein tyrosine phosphatase inhibitor vanadate on fusion–related proteins of myogenic cells. Ric10 cells were cultured for 24 h in pmDM and then cultured in pmDM supplemented with 0.1% DMSO (−) or SU6656 (+) for 9 h (A), or vanadate (1, 10, 100 μM) for 24 h (B). Total lysates (20 μg of proteins) were subjected to immunoblot analyses. MyHC, myosin heavy chain; p120PY228, tyrosine phosphorylated p120; p120PT310, threonine phosphorylated p120. β-tubulin was used as a loading control. [file 1471-2121-14-37-S4.tiff]

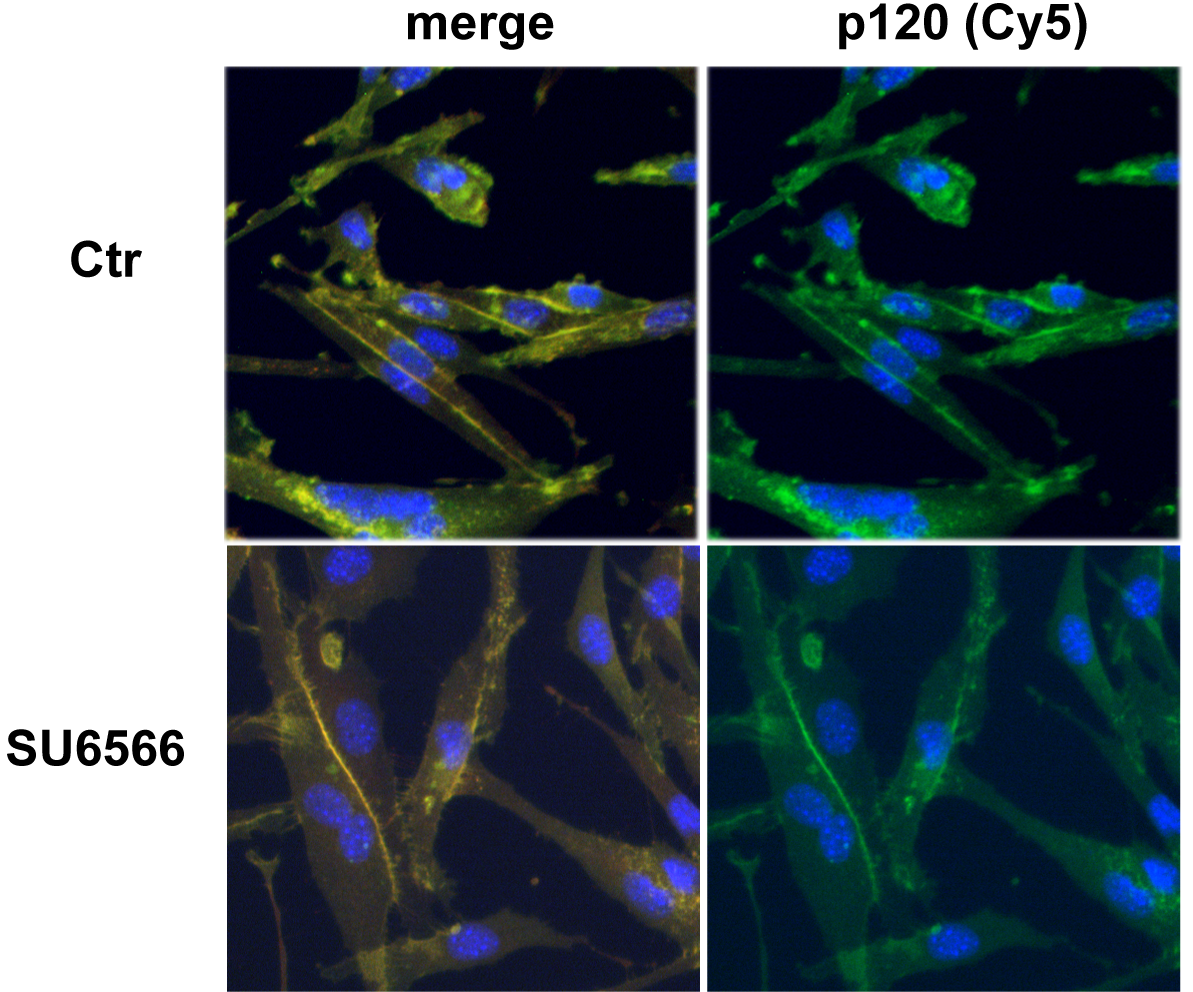

Supplement: Additional file 5 — Src kinase inhibitor doesn’t suppress accumulation of p120 at cell contacts. Ric10 cells were cultured for 24 h in pmDM and then cultured in pmDM supplemented with 0.1% DMSO (Ctrl) or SU6656 (100 μM) for 24 h. Tyrosine-phosphorylated p120 accumulated at cell-cell contacts in both control cultures (Ctrl) and SU6656- (green) and p120PY228 (red)-treated cultures. Cell nuclei were stained with DAPI (blue). Images were obtained by epifluorescence microscopy. [file 1471-2121-14-37-S5.tiff]
